# Adapting the Laser‐Induced Fluorescence Detection Setup of the Standard Capillary Electrophoresis Equipment to Achieve High‐Sensitivity Detection of 2‐Aminoacridone Labeled Oligosaccharides

**DOI:** 10.1002/jssc.70112

**Published:** 2025-03-16

**Authors:** Filip Dusa, Marcelina Rusin, Denisa Smolkova, Jozef Sestak, Justyna Dobrowolska‐Iwanek, Michał Woźniakiewicz, Jana Lavicka

**Affiliations:** ^1^ Institute of Analytical Chemistry of the Czech Academy of Sciences Brno Czech Republic; ^2^ Doctoral School of Exact and Natural Sciences Jagiellonian University Kraków Poland; ^3^ Department of Analytical Chemistry Faculty of Chemistry Jagiellonian University Kraków Poland; ^4^ Department of Chemistry Faculty of Science Masaryk University Brno Czech Republic; ^5^ Department of Food Chemistry and Nutrition Faculty of Pharmacy Jagiellonian University Medical College Kraków Poland

**Keywords:** 2‐aminoacridone, capillary electrophoresis, human milk oligosaccharides, laser‐induced fluorescence

## Abstract

The high‐sensitivity capabilities of laser‐induced fluorescence (LIF) detection continuously promote the development of various labels with different fluorescence properties. However, this strategy also requires the adaptation of existing detection systems to suit the excitation and emission characteristics of novel fluorescent tags. In this study, we adapted the LIF detector of the commercial capillary electrophoresis instrument to the specific fluorescence spectra of 2‐aminoacridone labeled human milk oligosaccharides. An external solid‐state laser with a wavelength of 405 nm was connected to the commercial capillary electrophoresis instrument via a simple 3D‐printed laser‐to‐light‐guide adapter, and different optical filter setups were compared based on the signal‐to‐noise ratio. The optimized setup provided detection limits as low as 0.27 to 0.34 nM, corresponding to injection of 3.4 to 4.6 attomoles of 2‐aminoacridone labeled oligosaccharides. These findings show that the optimized laser and filter configuration can enhance the sensitivity of electrophoretic separation by several orders of magnitude. In addition, the presented setup can be utilized as a guide for coupling different lasers to the commercial instrument.

**Abbreviations**
2′FL: 2′‐fucosyllactose
2‐AMAC: 2‐aminoacridone
3‐FL: 3‐fucosyllactose
3′SL: 3′sialyllactose
6′SL: 6′sialyllactose
APTS: aminopyrenetrisulfonate
BGE: background electrolyte
CE: capillary electrophoresis
DSLNT: disialyllacto‐*N*‐tetraose
HMOs: human milk oligosaccharides
ID: inner diameter
LIF: laser‐induced fluorescence
LNDFH I: lacto‐*N*‐difucohexaose I
LNDFH II: lacto‐*N*‐difucohexaose II
LNFP I: lacto‐*N*‐fucopentaose I
LNnT: lacto‐*N*‐neotetraose
LNT: lacto‐*N*‐tetraose
OD: optical density

## 1 Introduction

Fluorescence detectors can provide high sensitivity, superior to mass spectrometers or light‐absorbing spectrophotometry detectors [1]. The fluorescence sensitivity depends on both the fluorophore properties and the instrumentation used. From the instrumental point of view, the excitation source, as one of the essential parts of any fluorescence detector, should provide high‐intensity and stable output of a particular wavelength of light. In the case of capillary electrophoresis (CE), lasers, emitting very intensive, monochromatic, and coherent light, have become the dominant light sources, and CE with laser‐induced fluorescence detection (CE/LIF) represents a standard tool in various analytical fields, including glycomics.

Derivatization by a fluorescent label is a key step in the workflow of glycan and oligosaccharide analysis by CE/LIF [2]. Aminopyrenetrisulfonate (APTS) is the most commonly used fluorescent tag for glycan labeling followed by CE/LIF [3]. The excitation maximum of APTS‐labeled saccharides is at 455 nm, and a 488 nm laser is usually used for their excitation. The APTS‐labeled analytes exhibit strong fluorescence with an emission maximum at 512 nm, and a 520 nm bandpass filter is typically employed in the detector. This analytical setup can reach the picomolar limit of detection. Several other fluorescent labels have been introduced for oligosaccharide and glycan analysis [2]. However, they usually suffer from lower detection sensitivity compared to APTS. For example, 2‐aminoacridone (2‐AMAC) has been used for the analysis of glycosaminoglycans [4] and oligosaccharides [5, 6, 7, 8, 9, 10] by CE/LIF with nanomolar detection limits.

## 2 Protocol Motivation

**FIGURE 1:**
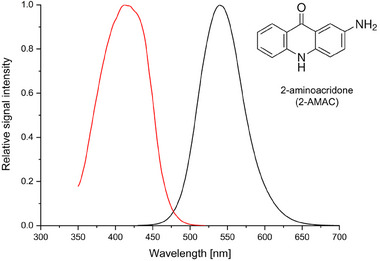
Excitation (red line) and emission (black line) spectra of 2‐AMAC measured in a 100 mM borate buffer, pH 10.5 (JASCO FP‐8500 fluorescence spectrometer, ABL&E‐JASCO, Vienna, Austria).

The commercially available CE/LIF systems are usually equipped with a 488 nm laser and thus the LIF detection of 2‐AMAC‐labeled analytes presented so far by other research groups was also obtained using this setup [4, 5, 6, 7, 8, 9, 10]. However, the excitation spectrum (Figure 1) indicates that significant sensitivity improvement in LIF detection of 2‐AMAC‐labeled compounds can be achieved by substituting the commonly used 488 nm laser by the laser with the emission line closer to the excitation maximum of the 2‐AMAC label (e.g., 43 times higher excitation efficiency using the 405 nm laser). Therefore, this protocol focuses on the adaptation of the LIF detection setup of the P/ACE MDQ Plus system (Sciex, Brea, CA, USA) by incorporating an external 405 nm solid‐state laser to achieve high‐sensitivity detection of CE/LIF analysis of 2‐AMAC‐labeled oligosaccharides in human colostrum and breast milk.

Note that:

• Data show that the excitation maximum of 2‐AMAC in the background electrolyte (BGE), 100 mM borate buffer, pH 10.5, is at 412 nm. This differs from the reported values (420–430 nm) used for fluorescence detection in HPLC analysis [11], which may be due to the different composition of mobile phases, that is 250 mM ammonium formate (pH 4.4) or 100 mM ammonium acetate (pH 6.6) in the acetonitrile gradient.

## 3 Chemicals and Materials

Lacto‐*N*‐difucohexaose I (LNDFH I), lacto‐*N*‐difucohexaose II (LNDFH II), lacto‐*N*‐neotetraose (LNnT), (lacto‐*N*‐fucopentaose I (LNFP I), 3′sialyllactose (3′SL), disialyllacto‐*N*‐tetraose (DSLNT), 3‐fucosyllactose (3‐FL), lacto‐*N*‐tetraose (LNT), 2′‐fucosyllactose (2′FL), and 6′sialyllactose (6′SL) were purchased from DextraUK and Biosynth Carbosynth (90%–99%). Lactose, 2‐AMAC, sodium cyanoborohydride, acetic acid, dimethyl sulfoxide, methanol, sodium hydroxide, hydrochloric acid, and sodium tetraborate decahydrate were purchased at the highest purity from Merck (Prague, Czech Republic).

Fused silica capillaries (50 µm inner diameter (ID), 365 µm outer diameter) were purchased from Molex (Lisle, IL, USA). Amicon filters (3 kDa MWCO) were purchased from Merck. Optical filters specified by part number (PN) in Section 4 were supplied by Edmund Optics Ltd (York, UK). A 405 nm solid‐state laser (RLDE405M‐50‐5) was purchased from Roithner (Vienna, Austria) and a 488 nm external laser (56‐CRN‐488‐050) was purchased from Melles Griot (Carlsbad, CA, USA). The fused silica ball lens, SMA bulkhead fiber adapter, and SMA–SMA solarization‐resistant MM fiber patch cable were purchased from Thorlabs (Newton, NJ, USA).

## 4 Technical Solution

**FIGURE 2:**
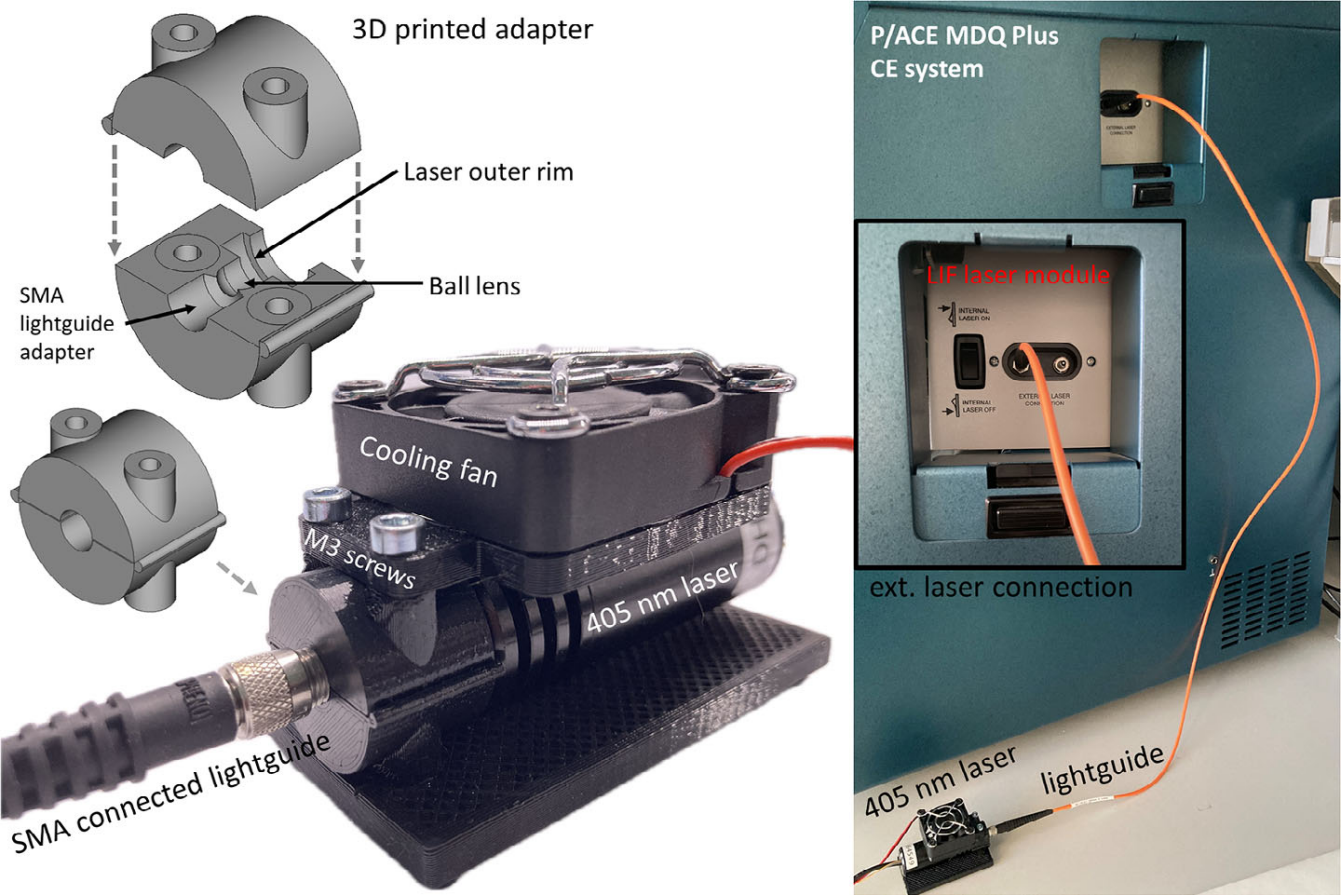
Scheme of the system and 3D‐printed adapter for laser connection. The adapter is composed of two identical parts that, when assembled, form a ball lens housing and an SMA coupling component. The upper platform is added as a fan holder, while the lower platform is used for additional stability, but these are not essential for the laser coupling setup.

The 405 nm solid‐state laser RLDE405M‐50‐5 (Roithner, Vienna, Austria) was connected to the SMA 905 input of the LIF module of the P/ACE MDQ Plus system via a 3D printed adapter and optical fiber as shown in Figure 2.

- The previously constructed simple circular laser adapter [12] was adapted to the specific outer rim of the 50 mW 405 nm laser.
- The laser‐to‐lightguide adapter was 3D‐modeled using open‐source software FreeCAD version 0.21.2. The 3D model file for printing (laser adapter 405 nm.3mf) is supplied in the Supporting Information.
- The adapter was designed to be printed as two identical halves with no supports needed during the printing process. The adapter was printed from black polyethylene terephthalate glycol 1.75 mm filament (EkoMB, Prague, Czech Republic) using a 3D printer MK3S+ (Prusa Research, Prague, Czech Republic).
- Assembly was done by settling the laser outer rim at the appropriate recess in one half of the adapter, the fused silica ball lens (diameter of 5 mm) was fitted in the middle of the adapter, and lastly, SMA bulkhead fiber adapter was fitted at the opposite side of the adapter.
- The second adapter half was added on the top and M3 screws (length 33 mm) with nuts were used to connect the adapter halves. An SMA–SMA solarization‐resistant MM fiber patch cable (1 m long, Ø200 µm, 0.22 numeric aperture, PN M112L01) was used to connect the adapter to the CE/LIF module.
- The 405 nm notch filter (OD 6, PN 86119) and 425 nm longpass filter (OD 4, PN 84736) or 500 nm longpass filter (OD 4, PN 62976) were installed in the filter holder of the LIF module of the P/ACE MDQ Plus system.

Note that:

• LIF module of the P/ACE MDQ Plus CE system includes the 488 nm laser. When it was used for comparative measurements, the 488 nm notch filter (Sciex) and 520 nm bandpass filter (Sciex) or 500 nm longpass filter (optical density (OD) 4, PN 62976) were installed in the filter holder.
• P/ACE MDQ Plus CE system is equipped with two channels for fluorescence detection, however, the second channel was not used in the experiments to avoid any bias related to the differences between the optical paths of the channels.

## 5 Oligosaccharide Labeling and CE Conditions

The test mixture of five HMOs consisted of lacto‐*N*‐difucohexaose II (LNDFH II), 3‐fucosyllactose (3‐FL), lacto‐*N*‐tetraose (LNT), 2′‐fucosyllactose (2′FL), and 6′sialyllactose (6′SL). The structures of individual HMOs are listed in Table S1 [13].

The 2‐AMAC labeling of HMOs was performed via a previously published procedure [10]. Briefly, HMO standards (1–2 nanomoles each) were labeled in a reaction mixture consisting of 25 µL of 25 mM 2‐AMAC (dissolved in a mixture of acetic acid:dimethyl sulfoxide (3:17, v/v)) and 25 µL of 1 M sodium cyanoborohydride (dissolved in water). The samples were incubated at 45°C for 2 h. Then, the labeled samples were diluted 10 or 100 times with dimethyl sulfoxide–water mixture (1:1, v/v) before CE/LIF analysis.

The samples were treated using the previously published procedure with some modifications [14]. Briefly, breast milk or colostrum (100 µL) was mixed with ultra‐pure water (100 µL). After stirring, samples were centrifuged at 6000 rpm, 4°C for 10 min. Avoiding the upper layer of fat, 100 µL of liquid was pipetted from the aqueous layer into a clean test tube. Then, acetonitrile (200 µL) was added, the sample was stirred, ultrasonicated for 5 min, and centrifuged at 10 000 rpm, 4°C for 10 min. The supernatant (250 µL) was taken, dried, and reconstituted in ultra‐pure water (450 µL). Finally, the samples containing HMOs were passed through the 3 kDa MWCO Amicon filters, and the flow‐through fractions were dried. The collected HMOs were derivatized with 2‐AMAC using the same procedure as HMO standards.

CE separations were performed in a fused silica capillary (50 µm ID, 50 cm effective length, 60 cm total length), BGE consisted of a 100 mM sodium borate buffer, pH 10.5, and 2‐AMAC‐labeled HMOs were separated as anionic borate‐polyol complexes [15]. The samples were injected into the separation capillary by applying a pressure of 0.5 psi for 15 s (∼ 13 nL), and CE separations were performed at 25°C by applying 15 kV separation voltage. The 32 Karat software was used for CE data acquisition and processing.

Note that, before each measurement day, the capillary was rinsed with 1 M sodium hydroxide for 10 min and with water for 5 min (with a pressure of 20 psi) to ensure cleaning of the inner surface and generation of consistent electroosmotic flow conditions. Due to this reason, the capillary was rinsed at 20 psi as follows: methanol for 1 min, 1 M hydrochloric acid for 2 min, water for 1 min, 1 M sodium hydroxide for 5 min, and background electrolyte (BGE) for 5 min between individual sample measurements.

## 6 Evaluation of the Adapted Setup Performance

The performance of the several LIF detection setups was compared based on the electropherograms of 2‐AMAC‐labeled HMO standards and corresponding signal‐to‐noise (S/N) ratios are summarized in Table 1. The highest S/N ratio was obtained using the LIF setup employing the 405 nm laser and the 500 nm longpass emission filter. A 43%–45% decrease in S/N ratios was observed using the 405 nm laser/425 nm longpass filter setup. Similar results were obtained with the LIF setup consisting of the 488 nm internal laser and 500 nm longpass filter. Despite our presumption, the 425 nm longpass filter provided lower S/N than the 500 nm longpass filter, which was caused by worse laser blocking resulting in higher background and noise. The lowest S/N ratios were obtained with the detection setup consisting of the internal 488 nm laser and 520 nm bandpass filter. This commercial LIF setup, which is commonly used in the published works, provided only 7% of S/N of the customized setup equipped with the external 405 nm laser/500 nm longpass filter combination.

**TABLE 1:** Comparison of the performance of the LIF setup for detecting 2‐AMAC‐labeled HMOs expressed in terms of S/N ratio (absolute values and relative values in %). The S/N ratio was calculated as the ratio of height of a particular peak to the baseline signal noise in front of the peak. Experimental conditions were the same as in Figure 3.

	S/N ratios (relative % values)			
	Original setup—internal laser		Customized setup—external laser	
HMOs	488 nm laser/520 nm bandpass filter	488 nm laser/500 nm longpass filter	405 nm laser/425 nm longpass filter	405 nm laser/500 nm longpass filter
LNDFH II	8.34 (7%)	67.31 (56%)	68.45 (57%)	119.62 (100%)
3‐FL	33.71 (7%)	259.40 (52%)	272.20 (55%)	495.60 (100%)
LNT	14.21 (7%)	123.63 (59%)	119.76 (57%)	208.47 (100%)
2′FL	17.87 (6%)	150.07 (49%)	177.04 (57%)	308.66 (100%)
6′SL	12.65 (6%)	117.61 (60%)	111.79 (57%)	195.75 (100%)

**FIGURE 3:**
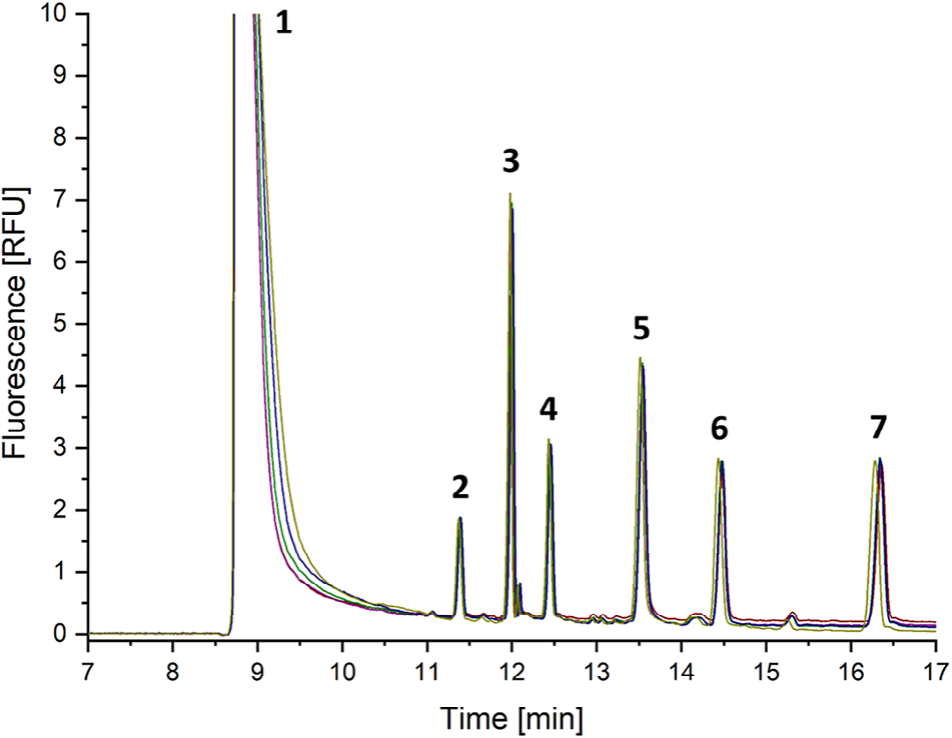
Overlay of 5 CE/LIF analyses of a test mixture consisting of five 2‐AMAC‐labeled HMOs. Experimental conditions are as follows: CE separation—50 µm ID capillary (50 cm effective length, 60 cm total length), BGE: 100 mM sodium borate buffer, pH 10.5, sample concentration: 2 µg/mL per each oligosaccharide (2.0–4.1 µM), sample injection: 0.5 psi, 15 s, separation voltage: 15 kV, LIF detection—excitation 405 nm laser, emission 500 nm longpass filter. Peaks: (1) 2‐AMAC, (2) LNDFH II, (3) 3‐FL, (4) LNT, (5) 2′FL, (6) 6′SL, and (7) unknown reaction product.

FIGURE 3 shows five repetitions of the CE/LIF analysis of 2‐AMAC‐labeled HMOs under the best‐performing detection conditions. This LIF setup provides the limits of detection of 0.27–0.35 nM (S/N = 3) and the limits of quantification of 0.89–1.18 nM (S/N = 10). In absolute values, the detection limits were determined to be 3.4–4.6 attomoles injected into the CE/LIF system. This corresponds to almost two orders of magnitude improvement in detection sensitivity compared to previously published data obtained using the LIF setup of the 488 nm laser and the 520 nm bandpass filter [4, 7, 10]. It should be mentioned that all the tested LIF setups provided repeatable results with the RSDs (*n* = 5) of peak heights and peak areas below 3.5% for all 2‐AMAC‐labeled HMOs.

## 7 Application of the Customized CE/LIF Setup for Analysis of Human Colostrum and Breast Milk Samples

**FIGURE 4:**
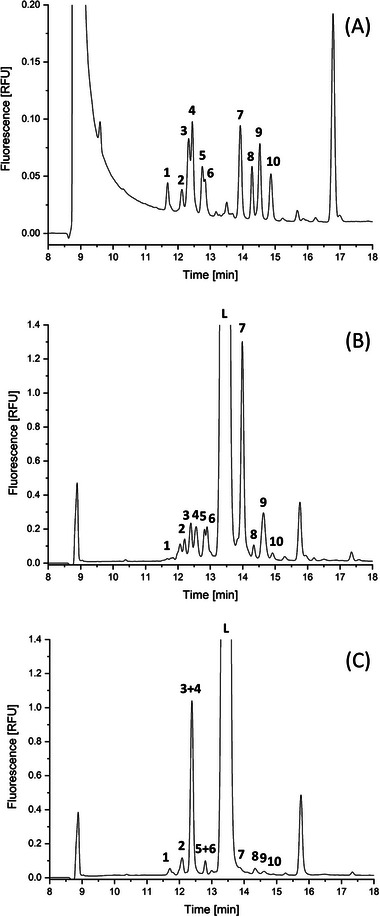
CE/LIF separation of 2‐AMAC‐labeled HMOs in (A) a test mixture of 10 HMO standards, (B) human colostrum, and (C) human breast milk. Experimental conditions are as follows: sample concentration: 0.1 µg/mL per each oligosaccharide standard, other conditions are specified in Figure 3. Peaks: (1) LNDFH II, (2) lacto‐*N*‐difucohexaose I (LNDFH I), (3) 3‐FL, (4) lacto‐*N*‐neotetraose (LNnT), (5) LNT, (6) lacto‐*N‐*fucopentaose I (LNFP I), (7) 2′FL, (8) 3′sialyllactose (3′SL), (9) disialyllacto‐*N*‐tetraose (DSLNT), (10) 6′SL, (L) lactose.

The optimized LIF detection setup (405 nm laser/500 nm longpass filter combination) was applied to CE/LIF analysis of HMOs in human colostrum and human breast milk. Samples were collected from patients at the University Hospital in Krakow, and after collection, the biological material was immediately frozen at −21°C. The study design was approved by the Bioethics Committee of the Jagiellonian University Medical College (No: 1072.6120.133.2018). The electropherograms obtained are shown in Figure 4B (human colostrum) and Figure 4C (human breast milk). Some HMOs present in colostrum and breast milk were identified based on the migration times of 2‐AMAC‐labeled HMOs standards analyzed under the same experimental conditions (Figure 4A).

## 8 Discussion of the Parameters Affecting the Effectiveness of the Customized LIF Detection Setup

Observed gain in S/N, when the standard internal 488 nm laser was substituted by the 405 nm external laser (Table 1) was much smaller than expected (2‐fold vs. 43‐fold expected). Based on the results that showed that the S/N with the 500 nm longpass filter was higher than with the 425 nm longpass filter, it appears that the quality (optical density) of the optical filters is one of the critical factors. The second factor is the amount of laser light power that truly enters the detection point of the capillary.

The overall power of 50 mW was in favor of the 405 nm external laser (vs. the 3 mW power output of the 488 nm internal laser), but it must be emphasized that tested lasers differ substantially in the coupling to the internal light guides which lead directly to the detection cell. The external laser has notably more connections, which causes dispersion and attenuation of the guided light. Therefore, a 50 mW 488 nm external laser was also coupled to the P/ACE MDQ Plus CE system via the 3D printed adapter and light guide as described above to compare the transmitted power of lasers used in this study. The power of light emitted from the light guides using both lasers was determined by Microscope Slide Thermal Sensor S175C (Thorlabs) connected to Power and Energy Meter Interface with USB Operation PM100USB (Thorlabs). The light‐guide output from the 405 nm laser was determined to be 8.4 mW and the 488 nm external laser outputted 1.4 mW from the light guide. The sixfold difference in output power is due to the non‐ideal coupling caused by the different designs of the 488 nm laser. The comparison of the acquired signals of 2‐AMAC‐labeled HMOs in relation to the power transmitted by the light guide is shown in Figure S1. The recorded fluorescence signal then differed by a ratio of 500. Thus, when accounting for the difference in output power (six times), the two orders of magnitude decrease in signal when using the 488 nm external laser confirms the low emission efficiency of the 2‐AMAC label at this wavelength. Under these experimental conditions, the benefits of using 405 nm laser expected based on an emission‐excitation spectrum of 2‐AMAC are clearly demonstrated. These results show that the light path from the internal laser to the detection cell is much better optimized than the light path for the external laser connection. While this feature of the LIF module cannot be easily changed, a more efficient coupling of the external laser to the external light guide can be utilized.

## 9 Concluding Remarks

Since the coupling of the lasers using only a ball lens was simple and varied slightly with different assembly trials, further optimization of the light‐guide coupling is planned in future studies to increase the total transmitted power. Nevertheless, this simplicity provides universal and easy setup which can probably surpass the unideal coupling parameters. Overall, the results show the utility of adjusting the LIF settings of commercial CE instruments based on the fluorescence characteristics of individual labels to improve the detection sensitivity of CE/LIF analyses. It should also be noted that the cost of the developed adaptation of the LIF detections setup (solid‐state laser module, laser power supply, cooling fan, optical filters, light guide, ball lens, SMA bulkhead adapter, and material for 3D‐printing) is estimated to about €1500, which is unbeatable compared to the commercial LIF module. The modified detection setup was demonstrated by analyses of HMOs in human colostrum and breast milk, which are usually present in relatively high concentrations. However, the mentioned adjusting of the CE/LIF is a relatively easy step not only to considerably increase the applicability of the 2‐AMAC‐labeling method but also to the analysis of the low‐concentrated oligosaccharides and glycans.

## Author Contributions

**Filip Dusa**: investigation, supervision, visualization, writing – original draft. **Marcelina Rusin**: investigation, formal analysis, writing – review and editing. **Denisa Smolkova**: investigation. **Jozef Sestak**: investigation. **Justyna Dowrowolska‐Iwanek**: writing – review and editing, resources. **Michał Woźniakiewicz**: writing – review and editing. **Jana Lavicka**: conceptualization, supervision, funding acquisition, project administration, writing – review and editing.

## Conflicts of Interest

The authors declare no conflicts of interest.

## Supporting information



Supplementary Material

Supplementary Material

## Data Availability

The data that support the findings of this study are openly available in the ASEP Data Repository at https://doi.org/10.57680/asep.0605745, reference number 0605745.
