# Supplementary material for: Adapting the Laser‐Induced Fluorescence Detection Setup of the Standard Capillary Electrophoresis Equipment to Achieve High‐Sensitivity Detection of 2‐Aminoacridone Labeled Oligosaccharides
Source: J Sep Sci. 2025 Mar 16;48(3):e70112. doi: 10.1002/jssc.70112 (PMC11910966; doi:10.1002/jssc.70112)
Supplement: Supplementary file 1 — Supplementary Material [file JSSC-48-e70112-s001.docx]

***Supplementary material***

**Adapting the standard CE equipment to achieve high-sensitivity detection of 2-aminoacridone-labeled oligosaccharides**

Filip Dusa^1^**^†^**, Marcelina Rusin^2,3^**^†^**, Denisa Smolkova^1,4^, Jozef Sestak^1^, Justyna Dobrowolska-Iwanek^5^, Michał Woźniakiewicz^3^, Jana Lavicka^1^

^1^ Institute of Analytical Chemistry of the Czech Academy of Sciences, Veveri 967/97, 602 00 Brno, Czech Republic

^2^ Doctoral School of Exact and Natural Sciences, Jagiellonian University, Prof. St. Łojasiewicza 11, 30‑348 Kraków, Poland

^3^ Department of Analytical Chemistry, Faculty of Chemistry, Jagiellonian University, Gronostajowa 2, 30‑387 Kraków, Poland

^4^ Department of Chemistry, Faculty of Science, Masaryk University, Kamenice 753/5, 625 00 Brno, Czech Republic

^5^ Department of Food Chemistry and Nutrition, Faculty of Pharmacy, Jagiellonian University Medical College, Medyczna 9, 31‑008 Kraków, Poland

**^†^** contributed equally.

*Correspondence should be addressed to the following author:

Dr. Jana Lavicka (orcid.org/0000-0002-0218-8372)

lavicka@iach.cz

**Table S1.** Structures of HMOs identified in human breast milk and colostrum.

| HMOs | | |
| --- | --- | --- |
| Name | Abbreviation | Structure |
| 2’-fucosyllactose | 2’FL | 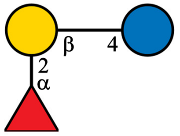 |
| 3’-fucosyllactose | 3’FL | 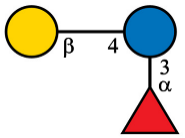 |
| 3’-sialyllactose | 3’SL | 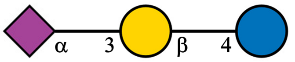 |
| 6’-sialyllactose | 6’SL | 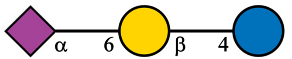 |
| lacto-*N*-tetraose | LNT | 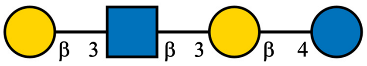 |
| lacto-*N*-neotetraose | LNnT | 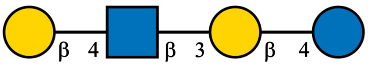 |
| lacto-*N*-fucopentaose I | LNFP I | 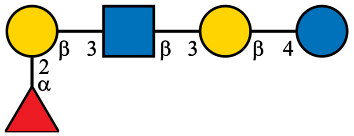 |
| lacto-*N*-difucohexaose I | LNDFH I | 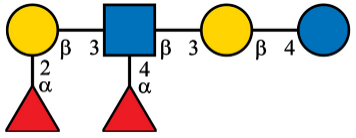 |
| lacto-*N*-difucohexaose II | LNDFH II | 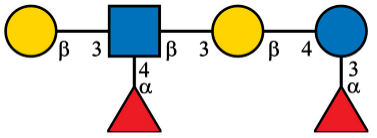 |
| disialyllacto-*N*-tetraose | DSLNT | 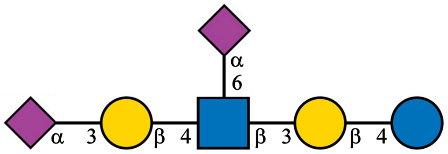 |

| 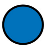 | glucose, | 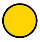 | galactose, | 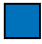 | *N*-acetylglucosamine, | 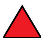 | fucose, | 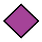 | *N*-acetylneuraminic acid |
| --- | --- | --- | --- | --- | --- | --- | --- | --- | --- |


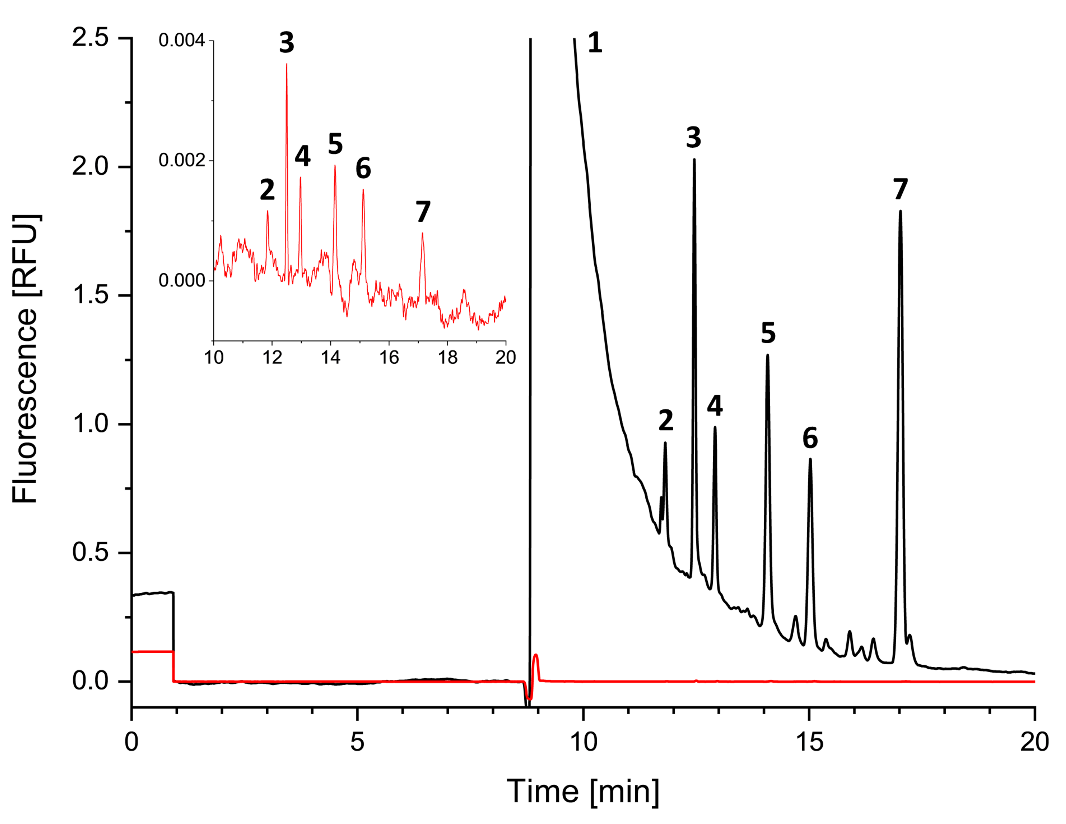


**Figure S1.** CE separations of 2-AMAC-labeled HMO standards detected by two 50 mW external lasers: black line - 405 nm laser (8.4 mW of output power in the lightguide), red line - 488 nm laser (1.4 mW of output power in the lightguide), emission 500 nm longpass filter; CE separation - 50 µm ID capillary (50 cm effective length, 60 cm total length), BGE: 100 mM sodium borate buffer, pH 10.5, sample concentration: 0.1 µg/mL per each oligosaccharide, sample injection: 0.5 psi, 15 s, separation voltage: 15 kV. Peaks: (1) 2-AMAC, (2) LNDFH II, (3) 3-FL, (4) LNT, (5) 2’FL, (6) 6’SL, (7) unknown reaction product. Inset – zoom of the signal obtained using the 488 nm external laser.
