# Supplementary figures and images for: Adapting the Laser‐Induced Fluorescence Detection Setup of the Standard Capillary Electrophoresis Equipment to Achieve High‐Sensitivity Detection of 2‐Aminoacridone Labeled Oligosaccharides
Source: J Sep Sci. 2025 Mar 16;48(3):e70112. doi: 10.1002/jssc.70112 (PMC11910966; doi:10.1002/jssc.70112)

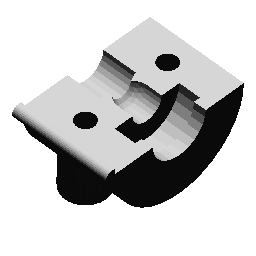

Supplement: Supplementary file 2 — Supplementary Material [file JSSC-48-e70112-s002.3mf › Metadata/thumbnail.png]
